# Simultaneous excitation and emission enhancements in upconversion luminescence using plasmonic double-resonant gold nanorods

**DOI:** 10.1038/srep15235

**Published:** 2015-10-15

**Authors:** Xin Liu, Dang Yuan Lei

**Affiliations:** 1 Department of Applied Physics, The Hong Kong Polytechnic University, Hong Kong, China; 2 Key Lab of Advanced Transducers and Intelligent Control System of Ministry of Education and Shanxi Province, College of Physics and Optoelectronics, Taiyuan University of Technology, Taiyuan 030024, China

## Abstract

The geometry and dimension of a gold nanorod (GNR) are optimally designed to enhance the fluorescence intensity of a lanthanide-doped upconversion nanocrystal placed in close proximity to the GNR. A systematic study of the electromagnetic interaction between the upconversion emitter of three energy levels and the GNR shows that the enhancement effect arising from localized electric field-induced absorption can be balanced by the negative effect of electronic transition from an intermediate state to the ground state of the emitter. The dependence of fluorescence enhancement on the emitter-GNR separation is investigated, and the results demonstrate a maximum enhancement factor of 120 folds and 160 folds at emission wavelengths 650 and 540 nm, respectively. This is achieved at the emitter-GNR separation ranging from 5 to 15 nm, depending on the initial quantum efficiency of the emitter. The modified upconversion luminescence behavior by adjusting the aspect ratio of the GNR and the relative position of the emitter indicates the dominate role of excitation process in the total fluorescence enhancement. These findings are of great importance for rationally designing composite nanostructures of metal nanoparticles and upconversion nanocrystals with maximized plasmonic enhancement for bioimaging and sensing applications.

Fluorescence imaging has been widely considered one of the most promising techniques in bioimaging because of its high spatial resolution, great sensitivity and low cost. In recent years, lanthanide-doped upconversion nanocrystals (UCNCs), which emit light at shorter wavelengths than the excitation wavelength, have emerged as excellent alternatives to organic fluorophores and quantum dots for fluorescence imaging. Compared to conventional counterparts, UCNCs exhibit many unique advantages such as non-photobleaching, weak photodamage to biological tissues, high fluorescence signal to noise ratio and large imaging penetration depth^1^,^2^,^3^,^4^,^5^,^6^,^7^. The upconversion refers to the nonlinear optical process that converts long-wavelength pump source into short-wavelength emission *via* successive absorption of two or more pump photons^8^. This nonlinear process in UCNCs makes use of the intermediate states of lanthanide ions (doped in inorganic crystals such as NaYF_4_ or CaF_2_) at which the excited state absorption (ESA) or non-radiative energy transfer (NRET) occurs for the generation of anti-Stokes emissions^9^. In addition to the inherent merits of large anti-Stokes shifts and sharp emission peaks, UCNCs also exhibit many other excellent properties such as long luminescence lifetimes, tunable multicolor emissions and low cytotoxicity. These unique properties of UCNCs make them significantly superior to organic fluorophores in, for example, bioimaging of small animals^10^,^11^,^12^,^13^,^14^ with significantly minimized photobleaching and unwanted non-specific fluorescence from live biological tissues when excited in the biologically transparent window at around 980 nm.

The distance between neighboring activator ions determined by their doping concentration and the absorption cross-section of the ions are the two major parameters that affect the upconversion efficiency. A low doping concentration of activator ions has to be kept in order to avoid quenching effect of excitation energy induced by the deleterious cross-section at high doping levels^15^,^16^, which consequently results in weak emission brightness. In addition, because of the intrinsic nature of anti-Stokes emission and low absorption cross-section of activator ions arising from the physically forbidden f-level atomic transitions of the dopants^8^, the absolute quantum efficiency is generally on the order of 1% and even lower^17^,^18^, depending on the excitation irradiance and emission wavelength of UCNCs. Besides the recent studies dedicated in accurate control of the morphology^19^, phase^20^,^21^, and emission colors of UCNCs^15^,^22^, it still remains challenging to significantly improve the power conversion efficiency. More recently, it has been demonstrated that increasing excitation irradiance from 1.6 × 10^4 ^W/cm^2^ to 2.5 × 10^6^ W/cm^2^ can alleviate concentration quenching in upconversion luminescence, thus significantly enhancing the luminescence signal from NaYF_4_:Yb^3+^/Tm^3+^ by a factor of 70^23^. However, such high excitation radiance is clearly not favorable in fluorescence bioimaging because of the high-probability photodamage to biological tissues. The use of intense coherent excitation sources also loses the inherent advantages associated with upconversion processes, in comparison with conventional anti-Stokes emissions such as two-photon absorption and second harmonic generation for which either expensive pulsed lasers with high power density (10^6^ ~ 10^9^ W/cm^2^) or nonlinear optical materials with non-centrosymmetric lattice arrangement has to be used.

An alternative promising way, of interest here, relies on the possibility of strengthening the local electric field (E-field) intensity in the vicinity of an individual metallic nanostructure by a few orders of magnitude upon optical excitation of its localized surface plasmon resonances (LSPRs). The presence of a metallic nanostructure in close proximity to an UCNC can influence both its absorption in the near infrared (NIR) and emission in the visible, and thus has the possibility of enhancing the upconversion luminescence provided that the plasmon-induced fluorescence enhancement dominates over fluorescence quenching^24^. A few studies have conclusively pointed out a positive relationship between the off-resonance excitation and the enhanced upconversion luminescence in different plasmonic metal-coated upconversion nanostructures. However, these studies often focused on the enhancement of upconversion luminescence by adjusting the plasmon resonance position of metal nanostructures to match the emission wavelengths of UCNCs without taking into account the effect of on-resonance excitation or absorption in an upconversion process^25^,^26^,^27^. Although a recent study has reported overlapping of a broad LSPR band of a gold nanoshell with the absorption of UCNCs in order to take advantage of the enhanced E-field around the nanoshell, a relatively low total fluorescence enhancement factor of ~2.6 folds was achieved due mainly to the off-resonance excitation^28^. On the other hand, a recent study designed and fabricated a silver nano-grating on which three monolayers of UCNCs were deposited^29^: The hybrid structure had a LSPR band at the absorption wavelength of the UCNCs, which consequently generated ~16 and ~39 folds enhancement in the green and red luminescence intensities. More recently, two experimental studies investigated the enhancement of upconversion luminescence by tailoring the LSPR wavelengths of GNRs^30^ and silver nano-platelets^31^ to match the excitation wavelengths of UCNCs. To the best of our knowledge, the moderate enhancement in upconversion luminescence intensity can be attributed to fact that the plasmon resonance wavelengths of metallic nanostructures used in all previous studies matched either the emission or absorption wavelengths of UCNCs. Distinctively different from these studies, here we propose to use a double-resonant GNR to match its two plasmon resonance bands with both excitation and emission wavelengths of UCNCs in order to maximize the enhancement of upconversion luminescence. The double resonant property of the GNR, namely two transverse and longitudinal LSPR bands, allows for simultaneous enhancement of both excitation and emission rates of a nearby emitter. In this work, we present a theoretical study of the upconversion luminescence enhancement of a dipolar upconversion emitter as functions of the distance from and relative arrangement to a GNR and distinguish the respective contributions from on-resonance excitation, spontaneous transition from an intermediate state to ground state, and emission enhancement. We show that the overall fluorescence enhancement can be maximized by optimizing the emitter-GNR separation distance and adjusting the GNR aspect ratio.

## Results

### Theoretical considerations of plasmon-enhanced upconversion luminescence

**Figure 1:**
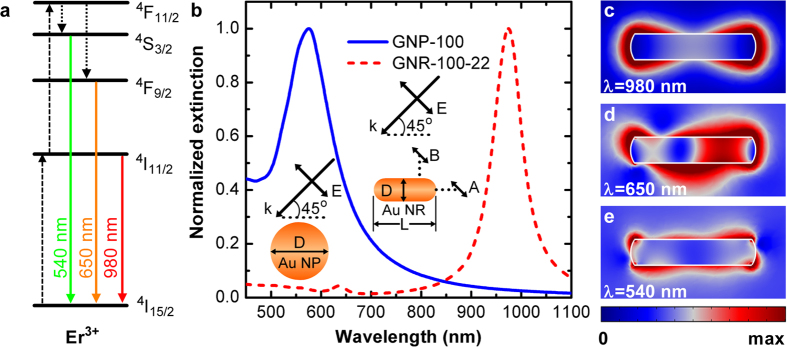
Simplified energy-level scheme of Er^3+^ doped UCNCs and plasmonic properties of gold nanostructures. (**a**) Simplified energy-level scheme of Er^3+^ doped UCNCs. Dashed, dotted and solid arrows indicate photon excitation, multi-phonon relaxation and emission processes, respectively. (**b**) Normalized extinction spectra of a gold nanoparticle with 100-nm diameter (blue curve) and a gold nanorod with 22-nm diameter and 100-nm length (red dashed curve). The insets in (**b**) show the excitation configuration for the gold nanoparticle and nanorod. Position A is on the longitudinal axis and position B is above the nanorod at a horizontal distance a quarter of the nanorod length to the nanorod end (**c**–**e**) Normalized E-field distribution in the GNR at wavelengths 980 (**c**), 650 (**d**), and 540 nm (**e**), corresponding to the excitation and emission wavelengths in (**a**).

Previous experimental results have demonstrated a substantial plasmon-induced upconversion luminescence enhancement^32^ much higher than theoretical prediction^33^ that is around unity for a co-doped UCNC with a pure NRET-mediated upconversion process. The discrepancy can be attributed to the fact that the complexity of the energy level system of the UCNC may have not been fully considered in the theoretical model, and that the ESA-mediated upconversion mechanism cannot be simply excluded under confocal excitation configuration with relatively high pump intensity. Thus, our work focuses on studying plasmon-enhanced upconversion luminescence *via* an ESA-mediated process and designing double-resonant GNRs to match both visible-wavelength upconversion emission peaks and NIR excitation wavelength. In the ESA-mediated upconversion process, one could expect the proportionality between upconversion signal and localized E-field amplitude enhancement to be similar to the well-known fourth power relationship valid for surface-enhanced Raman scattering. The actual enhancement factor of a coupled plasmonic nanoparticle-UCNC system is, however, far from that guaranteed by this law because the ESA process differs essentially from the Raman process. Specifically, although the forth power dependence in the upconversion luminescence can be well accounted by a two-photon absorption process, the decaying from the intermediate energy state to the ground state generates a negative effect (see Fig. 1a) to the whole upconversion process. In the weak excitation regime without reaching saturation and assuming that the environment does not affect the polarizability of the emitter, the excitation enhancement of an upconversion emitter nearby a GNR can be expressed as^33^


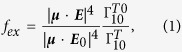


where ***μ*** is the transition electric dipole moment, and ***E***_0_ and ***E*** are the incident field and localized E-field under excitation of LSPRs, respectively. The total decay rate 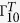 from the intermediate state 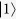 to the ground state 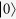 (^4^I_11/2_ to ^4^I_15/2_ for Er^3+^, see Fig. 1a) is the sum of three contributions 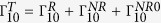 that represent respectively the radiative decay rate (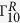) resulting in photon emission and the non-radiative decay rates including energy dissipated to the metallic nanostructure (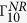) and energy to the internal decay channels of the emitter (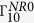). 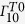 and 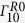 represent the corresponding quantities in a homogenous medium in absence of the metallic nanostructure while the internal decay channels (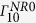) remain the same in both cases. One can clearly see from equation (1) that the excitation enhancement includes two competing contributions, namely the positive contribution from the fourth power of near-field enhancement 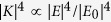 (ESA enhancement factor) and the negative contribution from the total decay rate of the intermediate state inversely proportional to the ratio 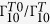 (relaxation factor).

For an isolated emitter, the initial quantum efficiency for the emission transition from the excited state 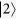 to the ground state 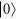 (^4^S_3/2_ to ^4^I_15/2_ or ^4^F_9/2_ to ^4^I_15/2_ for Er^3+^, see Fig. 1a) can be expressed as 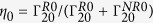 with 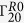 being the radiative decay rate and 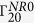 being the non-radiative decay rate (related to the intrinsic loss of the emitter). When the emitter is placed in the vicinity of a metallic nanostructure (also referred to as an optical antenna), the total decay rate changes to 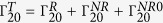, where 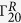 is the antenna-accelerated radiative decay rate of the emitter and 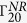 represents the non-radiative decay rate due to energy dissipated to the metallic nanostructure. The ratio 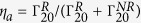 is defined as the antenna efficiency of the metal nanostructure. Then the Purcell factor *F*, which is the radiative decay rate enhancement, can be expressed as 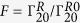. Since the antenna-modified quantum efficiency *η*(*ω*) is related to the original quantum efficiency *η*_0_(*ω*), the Purcell factor *F*(*ω*) and the antenna efficiency *η*_*a*_(*ω*) at emission frequency *ω*, the emission enhancement of the emitter can be calculated according to^34^


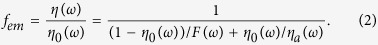


Thus, the total fluorescence enhancement of the upconversion emitter can be considered as the product of excitation enhancement and quantum efficiency enhancement^35^


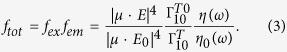


In our study, the three-dimensional finite-difference time-domain (3D-FDTD) method was adopted to calculate the radiative property of a single point dipole source, representing a single UCNC in what follows, at variable distance from a freestanding GNR. This method has been widely applied in the calculation of decay rates of fluorescent molecules near metallic nanostructures or in dielectric environment^36^, and a thorough proof of the equivalence between the quantum and classical results and the general methodology for calculating the decay rates with this method have been given^37^. In the electromagnetic treatment, the decay rates are proportional to the corresponding power ratios, which means all the quantities can be computed by considering the power emitted by a classical oscillating dipole close to an optical antenna and normalizing it with respect to the case without the optical antenna. We define that 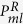 is the power reaches the far field and 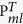 is the total power emitted by the dipole source, including the power dissipated to the metal. Here, the subscripts represent the electric transitions from 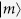 to 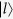, *m* = 1, 2 and *l* = 0. The quantities can then be given as 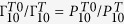, 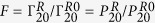 and 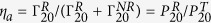, where the symbols superscripted with 0 correspond to the quantities for the isolated emitter in uniform background medium. Here 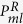 and 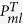 are the powers collected by integrating respectively the Poynting vector over an outer surface enclosing the emitter-antenna system and an inner surface only enclosing dipole emitter.

### Geometry dependence of GNR LSPRs

In our study, we choose an Er^3+^-doped UCNC as a model upconversion emitter because its visible-wavelength emission has great potential application in clinical biomedical research. Under laser excitation at 980 nm, it usually exhibits two main emission peaks at 540 nm (^4^S_3/2_ to ^4^I_15/2_) and 650 nm (^4^F_9/2_ to ^4^I_15/2_)^38^. Figure 1a shows the simplified schematic energy levels for Er^3+^, in which there are supposed to be many vibrational levels (not shown) around each electronic energy level. In general, it is reasonable to ignore these vibration relaxation processes because they are much faster than that between two electronic energy levels. Since the excitation and emission of the upconversion emitter are two independent processes, the changes in local density of states at each energy with the presence of a GNR can be treated separately through classical electromagnetic methods^39^.

Previous studies have shown that the longitudinal LSPR wavelength of an GNR exhibits an almost-linear red-shift with increasing the GNR’s aspect ratio while the transverse resonance wavelength shows little dependence on the aspect ratio^40^. In addition, the LSPR excitation in a GNR is highly polarization dependent due to its strong anisotropic response. By carefully taking into account these geometry and polarization dependences, the matching of the LSPR bands of a GNR with both absorption and emission wavelengths of an upconversion emitter could be achieved, which is superior to the use of a spherical gold nanoparticle (GNP) in improving the upconversion luminescence. For comparison, we calculated the normalized extinction spectra of an Au nanosphere and a GNR both immersed in water using the 3D-FDTD method. The length of the GNR is fixed to 100 nm and its longitudinal LSPR wavelength is tuned nearly to 980 nm (corresponding to the absorption peak wavelength of Er^3+^ indicated in Fig. 1a) by simply adjusting the GNR diameter to 22 nm. The calculation results and geometry configurations are shown in Fig. 1b. It can be found that the LSPR peak of the GNP “GNP-100” (denoting a spherical GNP of 100-nm diameter) is around 540 nm, which only overlaps with the emission peaks of Er^3+^. In contrast, on the one hand, the longitudinal LSPR peak of the GNR “GNR-100-22” (denoting a GNR of 100-nm length and 22-nm diameter) is at 980 nm, corresponding to a dipole-like plasmon resonance as demonstrated by the E-field distribution in Fig. 1c, which exactly matches with the absorption wavelength of Er^3+^ and is expected to increase the absorption rate. In the short wavelength region, on the other hand, the quadrupole resonance of the GNR can be seen at 650 nm (as demonstrated by the E-field distribution in Fig. 1d) though its extinction intensity is weaker than the dipolar resonance. In addition, the E-field distribution at 540 nm (see Fig. 1e) reveals a very weak response of the transverse LSPR of the GNR. Thus, the quadrupolar plasmon resonance and the transverse mode cover the whole emission wavelengths of Er^3+^, which can consequently affect the quantum efficiency enhancement at the two emission wavelengths^41^.

### Emitter-GNR separation dependence of upconversion luminescence enhancement

As shown in equation (3), the total enhancement of upconversion luminescence can be ascribed to two parts of contributions, excitation enhancement at the pump wavelength and the quantum efficiency enhancement (i.e. emission enhancement) at the emission wavelength. While the former effect is mainly determined by the local E-field intensity, the latter one has a complex dependence on the Purcell factor, the antenna efficiency and the initial quantum efficiency of the emitter as indicated by equation (2). To achieve efficient luminescence enhancement from an upconversion emitter, ideally, all the factors have to be increased at an appropriate dipole-antenna separation. As predicted in previous studies^42^,^43^, unfortunately, these conditions cannot be satisfied simultaneously at the same frequency. For instance, the high antenna efficiency usually means a faster radiative decay rate, which can reduce both the quality factor and the E-field enhancement. Thus, it is necessary to perform systematic calculations to determine the optimal conditions for the coupled emitter-GNR system by balancing several critical geometrical parameters, such as GNR aspect ratio, emitter-GNR distance and emitter orientation.

**Figure 2:**
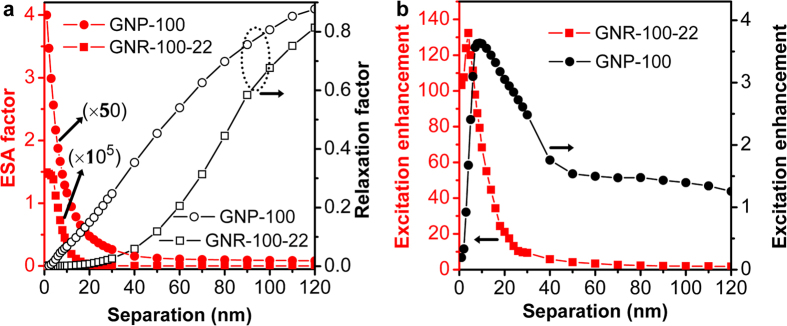
Excitation enhancement upconversion process with ESA mechanism for GNR. (**a**) The fourth power of local E-field–induced ESA factor (red) and relaxation factor (black) as a function of separation for GNR (squares) and GNP (circles). (**b**) Excitation enhancement as a function of separation for GNR (red squares) and GNP (black dots). The calculation of excitation is performed at wavelength of 980 nm under the configuration in Fig. 1b with dipole emitter located at position A.

In this work, we first consider a UCNC located at the end of a GNR (position A) and oriented along the incident light polarization as sketched in the inset of Fig. 1b, with the emitter-GNR separation distance ranging from 2 nm to 120 nm. Here, the emitter-GNR separation distance is defined as the distance between the emitter and the surface of the GNR. Figure 2a compares the ESA enhancement factor |*K*|^4^ arising from the fourth power of the near-field enhancement with the relaxation factor due to the spontaneous transition from intermediate state to ground state as a function of the emitter-GNR separation distance for GNR-100-22 and GNP-100. At small separation distances (<20 nm), it is found that the ESA enhancement factor for GNR-100-22 is almost three orders of magnitude larger than that for GNP-100 while their relaxation factors are comparable. This comparison points out the great promise of using the GNR for efficiently increasing the excitation efficiency of the upconversion emitter, resulting from the on-resonance excitation of the emitter at 980 nm. This indication is further corroborated by the overall excitation enhancement for both structures as shown in Fig. 2b, where the enhancement factor for GNR-100-22 is significantly larger than that for GNP-100 and their factors reach a ratio up to 35 at separation distance of 4 nm.

**Figure 3:**
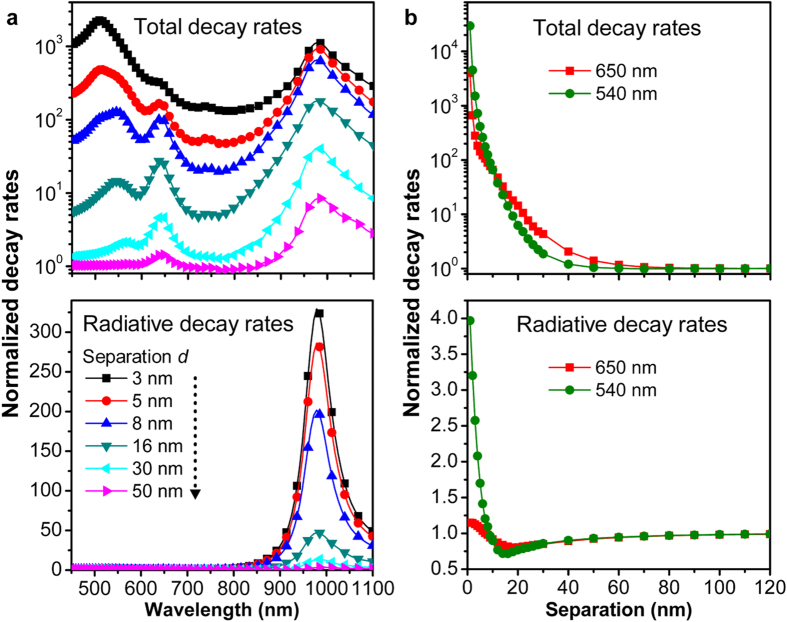
Normalized decay rates as functions of wavelength and separations. (**a**) Spectra of normalized total decay rates (upper) and radiative decay rates (bottom) for a dipole emitter coupled to a GNR (described in Fig. 1b) at different emitter-GNR separations. (**b**) Normalized total decay rates (upper) and radiative decay rates (bottom) at wavelength 650 nm (red squares) and 540 nm (green dots) as function of the emitter-GNR distance. The emitter is placed at position A in Fig. 1b.

Figure 3 shows the normalized total and radiative decay rates as functions of wavelength and separation. Figure 3a shows the two decay rates as a function of wavelength at emitter-GNR separation distances of 3, 5, 8, 16, 30, and 50 nm, which indicates strong modification to both decay rates at smaller distances due to stronger coupling of the dipole emission to the GNR plasmon resonance. More specifically, the upper panel of Fig. 3a shows that the normalized total power emitted by the dipole in the vicinity of the GNR exhibits three spectral peaks, corresponding to the dipolar, quadrupolar and transverse resonance bands of the GNR at 980, 650 and 540 nm, respectively. When the emitter is very close to the GNR tip (3-nm separation distance), the electromagnetic coupling between the emitter and the transverse plasmon resonance at 540 nm holds a prominent feature in the total decay rate, overwhelming the other two peaks. However, this coupling diminishes quickly as the separation distance increases, and the total decay rates at the quadrupolar and dipolar plasmon resonance bands (650 and 980 nm) decrease slowly for separation distance greater than 8 nm. In contrast, the lower panel of Fig. 3a shows that the normalized radiative decay rate (i. e. Purcell factor) is exclusively dominated by the longitudinal plasmon resonance at 980 nm for all separation distances, where the quadrupolar and transverse plasmon resonance bands have negligible modification to the radiative decay rate. Figure 3b shows the two decay rates as a function of separation distance at wavelengths 540 and 650 nm. On the one hand, the results shown in the upper panel indicate that the total decay rate at the two wavelengths can be enhanced by up to four orders of magnitude but decreases rapidly with increasing the separation distance. On the other hand, the lower panel of Fig. 3b shows that the radiative decay rates at 650 and 540 nm are not dramatically modified by the corresponding plasmon resonance bands, implying that the total decay rate enhancements are due mainly to the accelerated non-radiative decay rates leading to unwanted luminescence quenching. Although luminescence quenching due to Föster-type energy transfer and intrinsic absorption of the GNR at small separation distances dissipates a significant amount of emission energy at 540 and 650 nm, the strong scattering capability of the GNR can still direct the emission power to reach the far-field region. This is the basis for enhancing the total fluorescence of an UCNC with cooperation of local field-induced increased absorption.

**Figure 4:**
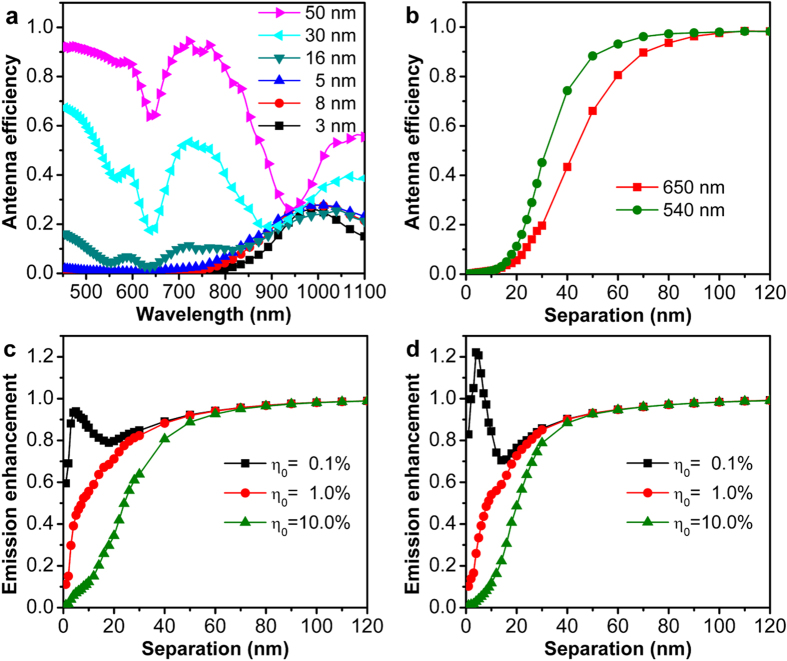
Antenna efficiency and emission enhancement of the emitter-GNR coupled system. (**a**) Spectrum of the antenna efficiency *η*_*a*_ for the emitter coupled to the GNR. (**b**) Antenna efficiency *η*_*a*_ at different wavelengths as a function of emitter-GNR separation. (**c,d**) Emission enhancement of the diploe as a function of emitter-GNR separation for different initial quantum efficiencies at emission wavelengths of 650 nm (**c**) and 540 nm (**d**), respectively. The calculation is performed using the configuration in Fig. 2.

As defined above, the antenna efficiency of a plasmonic nanostructure is the ratio of the radiative decay rate and the total decay rate for an emitter in the vicinity of the nanostructure. In the coupled emitter-GNR system, the antenna efficiency is calculated using the results presented in Fig. 3. One can see from Fig. 4a that the antenna efficiency is smaller than unity over the whole spectrum, consistent with previous predictions^33^, and increases as the emitter-GNR separation increases. Interestingly, for small separation distances (<16 nm), the maximum *η*_*a*_ appears in the spectral range of 900 nm to 1000 nm, resulting from the strong coupling between the emission from the dipole and the longitudinal plasmon resonance of the GNR because of the strong radiation property of the dipolar resonance. Surprisingly, for large separation distances (30 and 50 nm), *η*_*a*_ gets dominated in the short wavelength region and approaches to unity except the wavelength bands corresponding to the quadrupolar and transverse plasmon resonances. This is due to the fact that these two types of plasmon resonances usually have large Q factors and small radiation damping (i. e. weak scattering). Figure 4b shows that the antenna efficiency *η*_*a*_ dramatically increases with the emitter-GNR separation at the two emission wavelengths. As can be seen from equation (2), the emission enhancement also depends on the initial quantum efficiency of the emitter. Figure 4c,d show the emission enhancement for initial quantum efficiencies of 0.1%, 1.0% and 10.0% at the emission peaks of 650 nm and 540 nm, respectively. We can see from the results that, at both emission wavelengths, the emission enhancement for *η*_*a*_ = 0.1% is significantly larger than that for the other two larger quantum efficiencies at small emitter-GNR separation distances (<20 nm), with a maximum factor at 5-nm separation. This result is consistent with the prediction^34^ that when the *η*_0_ is close to 1, the dependence of emission enhancement on separation will follow the trend of *η*_*a*_ and can be only reduced. For the Purcell fcator is comparable to 1/*η*_0_, the emission enhancement depends both on *F* and *η*_*a*_. In this case, on the one hand, the competition between a small Purcell factor and *η*_*a*_ can only change the trend of quantum efficiency at small separations without obvious enhancement (e. g. the black squares for 650 nm in Fig. 4c). On the other hand, a strong Purcell factor can improve the quantum efficiency (e. g. the black squares for 540 nm in Fig. 4d), which can further contribute to the fluorescence enhancement at small separations.

**Figure 5:**
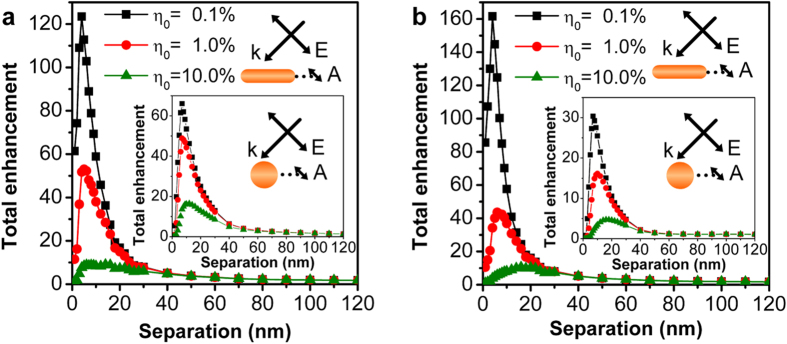
Total fluorescence enhancement of upconversion process with ESA mechanism for GNR and GNP (insets). Total fluorescence enhancement of the emitter-GNR coupled system for different initial quantum efficiencies *η*_0_ at emission wavelengths of 650 nm (**a**) and 540 nm (**b**), respectively. The calculation is performed using the configuration in Fig. 2. The insets in (**a**,**b**) are total enhancement corresponding to an emitter in the vicinity of gold GNP at emission wavelength of 650 nm and 540 nm, respectively.

Once we have obtained both excitation enhancement (see Fig. 2) and emission enhancement (see Fig. 4) for the coupled emitter-GNR system, we can now calculate the total fluorescence enhancement using equation (3). Figure 5a,b show the calculation results as a function of the emitter-GNR separation distance for the two emission wavelengths, respectively. It can be found that, for the emitter with *η*_0_ = 0.1%, the enhancement factor can be two orders of magnitude for both emission wavelengths at a separation distance of 5 nm. The total enhancement for both wavelengths is more than ten-folds even for relatively high initial quantum efficiency of *η*_0_ = 10% at separation distance of 10 ~ 15 nm. Similar results calculated for GNP-100 are shown as insets of Fig. 5. Due to the dual enhancement effect, the emitter-GNR system exhibits substantially larger total enhancement than the emitter-GNP system in all the situations except the emitter with *η*_0_ = 10% at the emission wavelength of 650 nm (see Fig. 5a and its inset).

### Emitter-GNR relative-location dependence of upconversion luminescence enhancement

**Figure 6:**
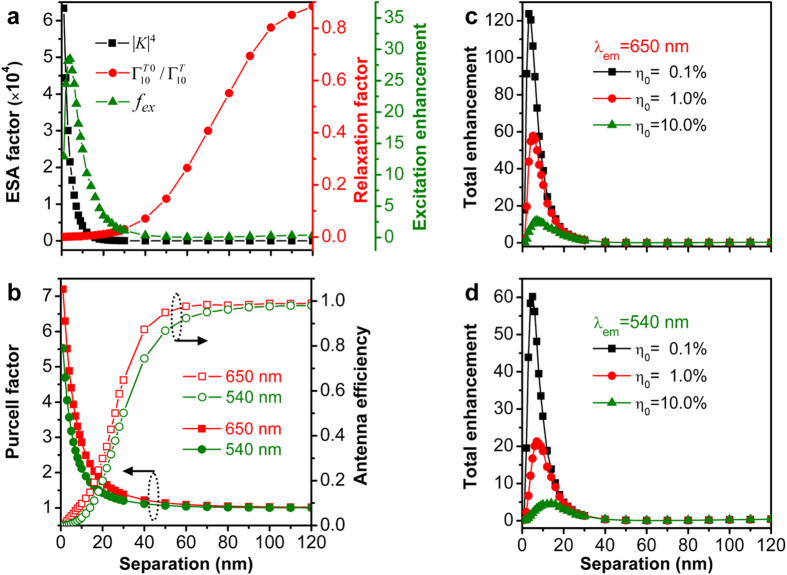
Position dependence of fluorescence enhancement of upconversion process. (**a**) The ESA factor (black squares), relaxation factor (red dots) and corresponding excitation enhancement (green triangles) as a function of the emitter-GNR separation. (**b**) Purcell factor *F* (solid) and antenna efficiency *η*_*a*_ (open) of the emitter coupled to GNR at position B indicated in Fig. 1b at emission wavelength of 650 nm (red) and 540 nm (green). (**c**,**d**) are the total fluorescence enhancement of the emitter-GNR coupled system for different initial quantum efficiencies *η*_0_ at emission wavelengths of 650 nm and 540 nm, respectively. The calculations are performed for excitation at wavelength of 980 nm.

The calculation results presented above are for the configuration in which the emitter is placed along the longitudinal axis of the GNR with the dipole orientation along the incident E-field polarization. For a practical UCNCs-coated GNR core-shell nanostructure, however, the relative orientation between the GNR core and the UCNC emitters is anisotropic, and therefore a strong location and polarization dependence of plasmon induced fluorescence enhancement was observed for a single GNR coated with dye molecules^44^. Since an UCNC placed at the lateral side of the GNR can only sense weak E-field enhancement, we have thus performed similar calculations by moving the dipole emitter to position B near the GNR as shown in the inset of Fig. 1b and keeping its orientation the same as the incident polarization. The excitation enhancement and its components, Purcell factor *F* and antenna efficiency *η*_*a*_, and the total fluorescence enhancement factor at the two emission wavelengths are plotted in Fig. 6. Figure 6a shows that the maximum excitation enhancement factor reaches 30-folds at separation distance of 5 nm, which is due to the joint contribution by the local field-enhanced excited state absorption (ESA factor) and the small transition rate from intermediate state to ground state (relaxation factor). This behavior is similar to that observed for position A as shown in Fig. 2 while the enhancement factor at position B is smaller. Figure 6b shows that the Purcell factor (antenna efficiency) at both emission wavelengths decreases (increases) with separation distance. In particular, the two quantities at emission wavelength of 650 nm are larger than at 540 nm, which can be ascribed to the fact that the emission at 650 nm from the emitter placed at position B can be more efficiently coupled to the quadrupole resonance of the GNR, thereby leading to a larger radiative decay rate. The total fluorescence enhancement factors at the two wavelengths are shown in Fig. 6c,d, respectively, which are very close to that for the emitter located at position A of the GNR. This is because the reduced excitation enhancement factor (due mainly to the smaller E-field enhancement) at position B is further compensated by the increased Purcell factor and antenna efficiency compared to position A. This result is particularly important for designing plasmonic nanorod core-UCNC shell structures because all the upconversion emitters can equally benefit from plasmonic enhancement effect regardless of their specific locations on the metal nanoparticle.

### GNR diameter dependence of upconversion luminescence enhancement

**Figure 7:**
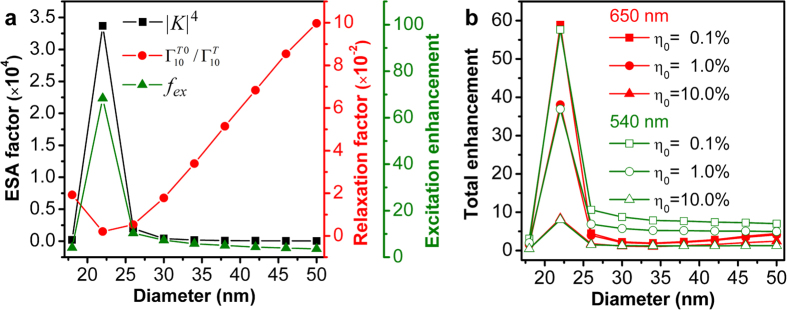
GNR diameter dependence of upconversion fluorescence enhancement. (**a**) Calculated ESA factor (black squares), relaxation factor (red circles) and corresponding excitation enhancement (green triangles) as a function of the diameter of a 100-nm-long GNR for a dipolar emitter placed at position A (see Fig. 1b) at 10 nm distance from the GNR. (**b**) Total fluorescence enhancement as a function the diameter for the same system as (**a**) at emission wavelengths 650 nm (red) and 540 nm (green) for UCNCs with different initial quantum yields.

It has been observed that the longitudinal and transverse plasmon resonance wavelengths of a GNR can be tailored by adjusting its aspect ratio. This indicates that variation in the diameter of a GNR with fixed longitudinal length can also have significant influence on the luminescence enhancement of an UCNC nearby the GNR. To explore this effect, we place a dipolar emitter at position A of a 100-nm-long GNR as shown in Fig. 1b, with an emitter-GNR separation distance of 10 nm when varying the GNR diameter from 18 nm to 50 nm in a step of 4 nm. The excitation enhancement and the total fluorescence enhancement are shown as a function of the GNR diameter in Fig. 7a,b, respectively. It can be clearly seen from Fig. 7a that the extremely enhanced local E-field dominates the excitation enhancement as manifested by very large ESA factors at small diameters. Although the relaxation factor has a minimum at the 22-nm diameter, the off-resonance excitation enhancement is slightly smaller than that of on-resonance configuration shown in Fig. 2b and follows the same trend as the ESA factor. In addition, Fig. 7b clearly shows a maximum total fluorescence enhancement occurring at the 22-nm diameter at both emission wavelengths 650 nm and 540 nm for UCNCs with different initial quantum efficiencies, demonstrating the dominate role of the excitation enhancement in the whole plasmon-enhanced upconversion processes. To this end, we have shown that the plasmon resonance dependent excitation and emission processes can be tailed by adjusting the aspect ratio of a GNR, allowing for sophisticated optimization of upconversion luminescence enhancement in experiment.

## Discussion

In the past few years, the integration of metallic nanoparticles and UCNCs has proven to be an efficient means to significantly enhance the upconversion luminescence efficiency, thereby overcoming the limit of low intrinsic quantum yields of UCNCs^17^,^18^. One of the major challenges related to this approach is to further enhance the upconversion luminescence efficiency by properly tuning the plasmon resonance bands and controlling the separation distance in between. To tackle this challenge, our work has theoretically evaluated the influence of these two factors in a hybrid system consisting of UCNCs and a double-resonant GNR that has two LSPR bands matching both the absorption and emission wavelengths of the UCNCs. We have found that both the spectral matching condition and the spatial separation distance have strong effects on the enhancement of upconversion luminescence. Indeed, our results have demonstrated that a significant enhancement of upconversion luminescence can be observed by precisely tuning the longitudinal (quadrupole and transverse) LSPR wavelength of the GNR at 980 nm (650 and 540 nm) in order to take advantage of the enhanced E-field and, at the same time, tuning the separation distance in the range from 5 to 15 nm for UCNCs with different initial quantum yields in order to overcome fluorescence quenching. The remarkable enhancement in the total luminescence results from both excitation and emission enhancements of the upconversion process.

In conclusion, we have performed a systematic numerical study to investigate the fluorescence enhancement of an Er^3+^-doped UCNC in the vicinity of a double-resonant GNR using the 3D-FDTD method. Our results demonstrate that when the LSPRs of GNR is tailored to match both the excitation and emission wavelegnth of UCNCs, the total fluorescence enhancement factor of the UCNC has a strong dependence on the emitter-nanorod separation distance and comes mainly from the local E-field induced excitation enhancement rather than the increased Purcell factor or the antenna efficiency of the nanostructre at emission wavelengths. The results demonstrate a maximum enhancement factor of ~120 folds and ~160 folds at emission wavelengths 650 and 540 nm, respectively, both of which are much larger than that reported for spherical metal nanostructures in previous studies. More importantly, the enhancement factor has little dependence on the relative position of the upconversion emitter on the gold nanorod. Consequently, we have showed that the maximum enhancement factor can be achieved at separation distance ranging from 5 to 15 nm, depending on the initial quantum efficiency of the upconversion emitter. Although our work is based on numerical calculations guided by sophisticated physical models, the findings from this study can still provide important guidelines for designing novel metal-nanoparticles-UCNCs composite nanostructures with significantly enhanced luminescence efficiency. For instance, the results of our work suggest that the separation distance between a GNR and UCNCs is a critical parameter for enhanced upconversion luminescence, and thus in experiment this distance should be precisely controlled by, for example, coating the GNR with polyelectrolyte multilayers or dielectric spacers. For potential biomedical imaging applications with high spatial resolution, the host material NaYF_4_ could be replaced by CaF_2_ with smaller sizes to facilitate attachment on the GNR with relatively larger dimensions via electrostatic attraction.

## Methods

We employed a commercial software package, FDTD Solutions, developed by Lumerical Solutions, Inc., to investigate the decay behavior of a dipole emitter in the vicinity of a GNR. In our calculations, the dielectric function of gold is modeled using a Drude-Lorentz dispersion model, and the refractive index of the surrounding medium is considered non-dispersive and equal to 1.33 for water. The simulation domain is enclosed by perfectly matched layers (PMLs) to absorb the outward propagating radiation. In the electromagnetic treatment, the far-field radiation power from the dipole source with and without the metallic antenna are collected by integrating the Poynting vector over the surface of a virtual box placed 600 nm away from the dipole. The total radiation power from the dipole is collected by integrating the Poynting vector over the whole surface of an inner small box that just encloses the dipole. In addition, the 3D-FDTD mesh discretization around the dipole is chosen to be 0.5 nm and a gradually increasing mesh size is used with a maximum mesh size of 20 nm for the region far from the coupled emitter-nanorod system.

## Additional Information

**How to cite this article**: Liu, X. and Yuan Lei, D. Simultaneous excitation and emission enhancements in upconversion luminescence using plasmonic double-resonant gold nanorods. *Sci. Rep.* **5**, 15235; doi: 10.1038/srep15235 (2015).
