# Erratum: Simultaneous excitation and emission enhancements in upconversion luminescence using plasmonic double-resonant gold nanorods

**DOI:** 10.1038/srep17121

**Published:** 2015-12-18

**Authors:** Xin Liu, Dang Yuan Lei

Scientific Reports 5 Article number: 15235; 10.1038/srep15235 published online: 10152015; updated: 12182015.

In the original version of this Article, Dang Yuan Lei was incorrectly listed as being affiliated with ‘Key Lab of Advanced Transducers and Intelligent Control System of Ministry of Education and Shanxi Province, College of Physics and Optoelectronics, Taiyuan University of Technology, Taiyuan 030024, China’.

In addition, Dang Yuan Lei should only be affiliated with ‘Department of Applied Physics, The Hong Kong Polytechnic University, Hong Kong, China’.

These errors have been corrected in both the HTML and PDF versions of the Article.

